# Passively Driven Probe Based on Miniaturized Propeller for Intravascular Optical Coherence Tomography

**DOI:** 10.1038/s41598-018-23547-4

**Published:** 2018-03-26

**Authors:** Yu Lu, Zhongliang Li, Nan Nan, Yang Bu, Xuebo Liu, Xiangdong Xu, Xuan Wang, Osami Sasaki, Xiangzhao Wang

**Affiliations:** 1 Laboratory of Information Optics and Opto-Electronic Technology, Shanghai Institute of Optics and Fine Mechanics, Chinese Academy of Sciences, Shanghai, 201800 China; 2 University of Chinese Academy of Sciences, Beijing, 100049 China; 3 Department of Cardiology, Tongji Hospital, Tongji University, Shanghai, 200065 China; 4 Cardiovascular department, Central hospital of Jiading District, Hospital affiliated to shanghai university of medicine and health sciences, Shanghai, 201800 China; 5 Faculty of Engineering, Niigata University, Niigata-shi, 9502181 Japan

## Abstract

Optical coherent tomography (OCT) has enabled clinical applications ranging from ophthalmology to cardiology that revolutionized *in vivo* medical diagnostics in the last few decades, and a variety of endoscopic probes have been developed in order to meet the needs of various endoscopic OCT imaging. We propose a passive driven intravascular optical coherent tomography (IV-OCT) probe in this paper. Instead of using any electrically driven scanning device, the probe makes use of the kinetic energy of the fluid that flushes away the blood during the intravascular optical coherence tomography imaging. The probe converts it into the rotational kinetic energy of the propeller, and the rotation of the rectangular prism mounted on the propeller shaft enables the scanning of the beam. The probe is low cost, and enables unobstructed stable circumferential scanning over 360 deg. The experimental results show that the probe scanning speed can exceed 100 rotations per second (rps). Spectral-domain OCT imaging of a phantom and porcine cardiac artery are demonstrated with axial resolution of 13.6 μm, lateral resolution of 22 μm, and sensitivity of 101.7 dB. We present technically the passively driven IV-OCT probe in full detail and discuss how to optimize the probe in further.

## Introduction

Optical coherence tomography (OCT) is an imaging modality for noninvasive medical diagnostics, and it enables cross-sectional and three-dimensional tomographic visualization of internal microstructure in biological systems^1^. In the last few decades, OCT has been used in clinical applications ranging from ophthalmology to cardiology, which revolutionized *in vivo* medical diagnostics^2^. In 1996, OCT was first applied for *in vitro* human vascular imaging to detect the atherosclerotic plaques^3^. Since then, in order to meet the needs of various endoscopic imaging, a variety of endoscopic probes have been developed by researchers^3–9^. Based on the relation between the propagation direction of the imaging beam and the longitudinal axis of the probe, OCT endoscope probes can be divided into side-viewing probes and forward-viewing probes, and also based on the location of the beam scanning device, they can be divided into proximal-end scanning probes and distal-end scanning probes^10^.

Basically, most of the probes have electric driven scanning device, and it can be called as “active driven” probe. The electrically driven scanning devices convert electrical energy into mechanical energy to scan the imaging beam. Commonly, in gastrointestinal OCT (GI-OCT) and intravascular OCT (IV-OCT)^11^, disposable catheter probes perform scanning by using external actuators and a drive shaft that relays torque and translation to optics mounted at the distal tip^12^. This kind of proximal-end scanning probes is susceptible to the image artifact referred to as non-uniform rotation distortion (NURD)^13^. NURD is a result of rotational friction of the hollow drive -shaft in the catheter, which leads to variable torque transfer from the proximal motor to the catheter tip^14,15^. In 2004, a distal-end side-viewing probe was achieved by installing a micro-motor at the end of the probe^8^. Such techniques have the advantage that kinks in the probe do not affect the rotational scan and the resultant images are much less susceptible to NURD than proximal-end scanning probes^13^. However, images suffer a wire-shadow effect^9^ because the micro-motor is mounted at the end of the probe, and this effect makes a portion of the images invisible. And the complex fabrication and fragility of the micro-motor makes miniaturization of the probe a challenging task. Moreover, the high cost of the micro-motor is a barrier for its clinical application. In 2013, Tianyuan Chen *et al*. presented a tiny endoscopic optical coherence tomography probe driven by a miniaturized hollow ultrasonic motor^9^. This kind of probe enables the fiber to pass through the inside of the hollow ultrasonic motor and makes it possible to have the fiber, the objective lens, and the motor on the same side, performing the imaging without any shadow. However, the component of this kind of probe is complicated and the maximum rotation speed of the hollow ultrasonic motor is only about 30 rotations per second (rps) under the driving voltage of 7 Vrms and current of 3 mA. The slow rotation speed of the probe limits the practical application of the probe.

In this article, we propose a novel passively driven IV-OCT probe based on a miniaturized propeller. Due to the blood is a strong attenuator of light, when IV-OCT imaging vessels, blood in the vessel needs to be removed. One way is injecting crystalloid or radiocontrast media into the vessel to flush away the blood when IV-OCT imaging is performing^16^. Another is using an occlusive intravascular balloon to interrupt blood flow, yet coronary occlusion carries a significant risk of ischemia and is not acceptable^13^. The fluid ejected from the gap between the inner sleeve and the outer sleeve can flush the blood, and we take advantage of the kinetic energy of the fluid when flushing the blood to rotate the propeller mounted at the end of the probe. A right-angle prism mounted on the propeller shaft is driven to rotate, and the light beam reflected by the prism can scan the vessel wall. Since this probe belongs to the distal-end side-viewing probe, the twisting problem is avoided, and the probe is less affected by NURD. The main feature of this probe is that it does not need an electrically driven scanning device, which leads to a low cost and shadow-free IV-OCT probe. In this study, we demonstrate the good performance of the probe which has an outer diameter of 3.5 mm and a rigid part length of 14 mm. The probe can reach scanning speed up to 176 rps.

## Results

### Probe design

**Figure 1:**
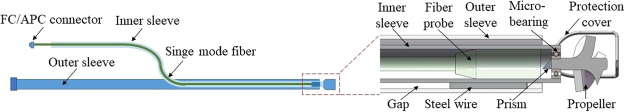
Schematic of the passive driven probe.

For this study, we adopt double-sleeve design for the probe. The schematic of the probe is shown in Fig. 1, where the probe mainly consists of inner sleeve, outer sleeve, optical fiber probe, micro bearing, protection cover, miniaturized propeller, right angle prism, and other components. The inner sleeve was inserted into the outer sleeve from the opening of the outer sleeve and extended beyond the end of the outer sleeve. The beam was transmitted from the FC/APC connector to the customized fiber probe. The fiber probe had a gradient refractive index (GRIN) lens at the end. The beam emitted from the GRIN lens was reflected by a right angle prism fixed to the propeller and then irradiated the lumen to be measured after passing through the inner sleeve. The working distance of the fiber probe was 3 mm with a beam waist of 22 μm. The fluid was injected from the outer sleeve, and then it flowed through the gap between the inner sleeve and the outer sleeve. This flowing fluid flushed away the blood and pushed the propeller to rotate. A larger gap can lead a greater fluid flow, thus the thrust of the propeller is greater and the rotation speed is faster. However, the larger gap will lead to a larger probe size while the size of the inner sleeve remains the same.

The size of the miniature bearing is critical because the sizes of the propeller and the probe are indirectly determined by the bearing size. As shown in Fig. 1, we used a micro bearing as a rotating part connecting the propeller and the inner sleeve. The micro bearing had an outer diameter (OD) of 1.5 mm and an inner diameter (ID) of 0.6 mm, and a width of 0.6 mm. The propeller had a diameter of 2.7 mm, an axial length of 4.1 mm, and a shaft diameter of 1 mm. The propeller shaft was mounted on the bearing bore, and the 0.5 mm right angle prism was mounted on the propeller shaft to reflect the light beam. The ID and OD of the inner sleeve were 1.5 mm and 1.9 mm respectively, in order to match the bearing size. And the ID and OD of the outer sleeve were 2.5 mm and 2.9 mm, respectively, in order to match the propeller size. Both the inner and outer sleeves are biocompatible fluorinated ethylene propylene (FEP) catheters. When the probe is working, only the micro bearing, the propeller, and the right angle prism rotate and the other parts of the probe remain stationary.

**Figure 2:**
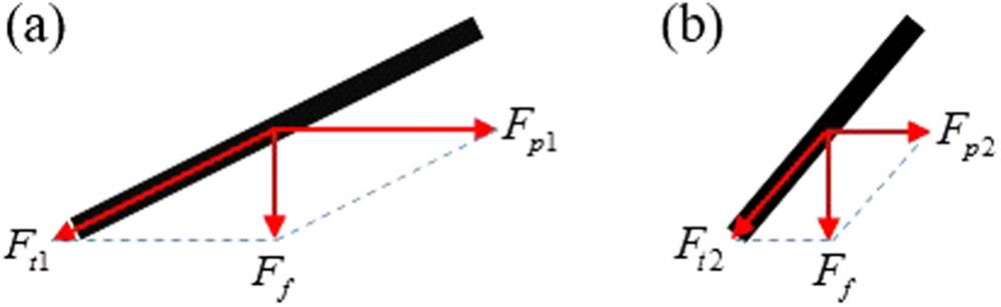
Force analysis of single blade with different slopes. *F*_*f*_ represents the thrust of the fluid subjected to the propeller. *F*_*pi*_ (*i* = 1, 2) and *F*_*ti*_ (*i* = 1, 2) represent the decompositions of the force *F*_*f*_ in the horizontal direction and the tilt direction, respectively. (**a**) a blade with a small tilt angle; (**b**) a blade with a larger tilt angle.

In the system, the propeller design is important, as it determines the energy conversion efficiency of the fluid. It is necessary to improve the efficiency in converting kinetic energy of the fluid into that of propeller rotation. In the last decade, a number of new tools have become available for propeller design^17^, but it is not an easy task even for an experienced designer to design a propeller by taking into account both high safety factors and high efficiency^18^. We simplified the propeller model to a single blade to make the analysis easier. The force analysis of the single blade with different slopes is shown in Fig. 2, where other conditions except the slope are the same.

As illustrated in Fig. 2, *F*_*pi*_ (*i* = 1, 2) is the force that drives the propeller to rotate, and *F*_*p*1_ >  *F*_*p*2_ in the case of the same *F*_*f*_. Therefore, the propeller with a smaller tilt angle of the blade will have a better conversion efficiency of hydrodynamic energy. However, for propellers with the same number of blades, the small tilt angle of the blades leads to small connecting part between the blade and the propeller shaft, which may cause the blades to be broken easily from the blade root. In the final design the tilt angle of the blade was about 44 deg, through a trade-off relation between the conversion efficiency and the safety factor.

**Figure 3:**
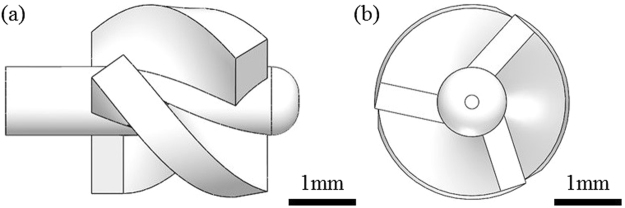
Design diagram of the miniaturized propeller. (**a**) Side view of the designed propeller; (**b**) Front view of the designed propeller.

The design diagram of the miniaturized propeller is shown in Fig. 3. 3D printing technology is a new manufacturing technique, which is developed rapidly in recent decades. It has the advantages of fast production and low production cost, and has been widely used in new products development, single-piece production and small-batch production. In this paper, we used the 3D printing technology to make the miniaturized propeller. The propeller shaft was produced slightly larger than the micro bearing bole, so the two parts are closely matched. Considering the micro bearing size and the accuracy of 3D printing, in the final design the propeller had a diameter of 2.7 mm, an axial length of 4.1 mm, and a propeller shaft diameter of 1 mm, and it has three blades with each thickness of 0.7 mm. The propeller and the probe were designed by SolidWorks.

**Figure 4:**
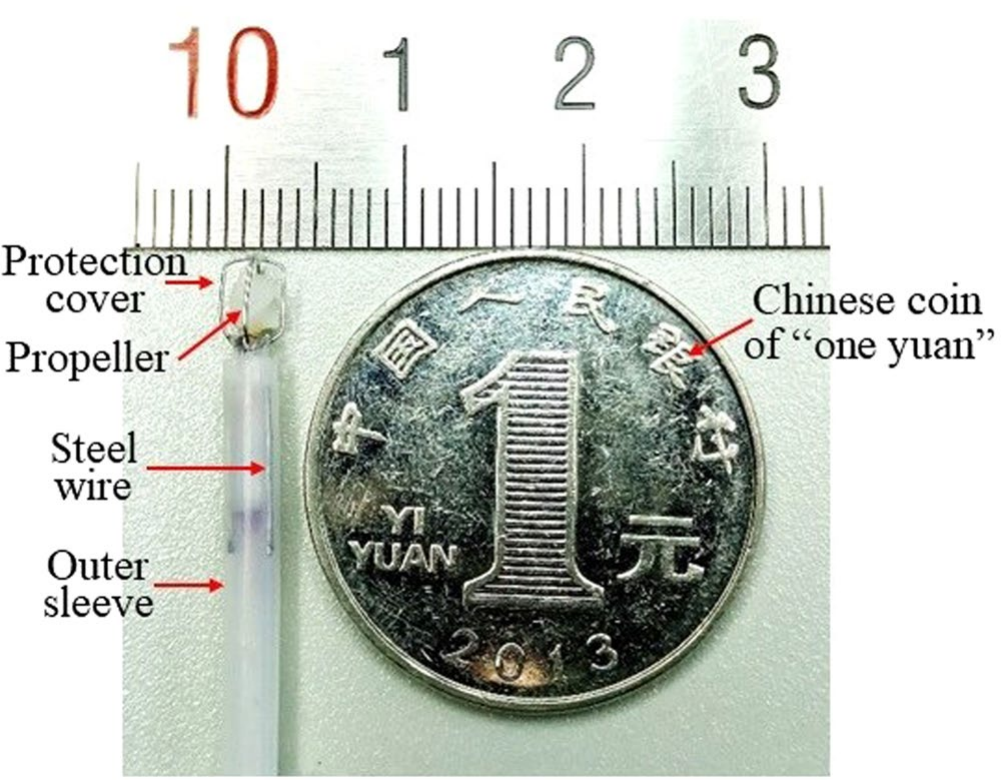
Photo of the probe with a maximal outer diameter of 3.5 mm.

A protection cover of stainless steel wire was designed to protect the propeller blade from contact with the lumen wall, as shown in Fig. 4. In addition, three stainless steel wires were used to ensure the coaxial of the inner and outer sleeve at the end. So the fluid ejected from a uniform cylindrical gap. This design resulted in the propeller rotating with uniform force. The end of the assembled probe is shown in Fig. 4, with a maximal diameter of 3.5 mm, a length of rigid part of 14 mm and a total length of 1.2 m.

### System design

**Figure 5:**
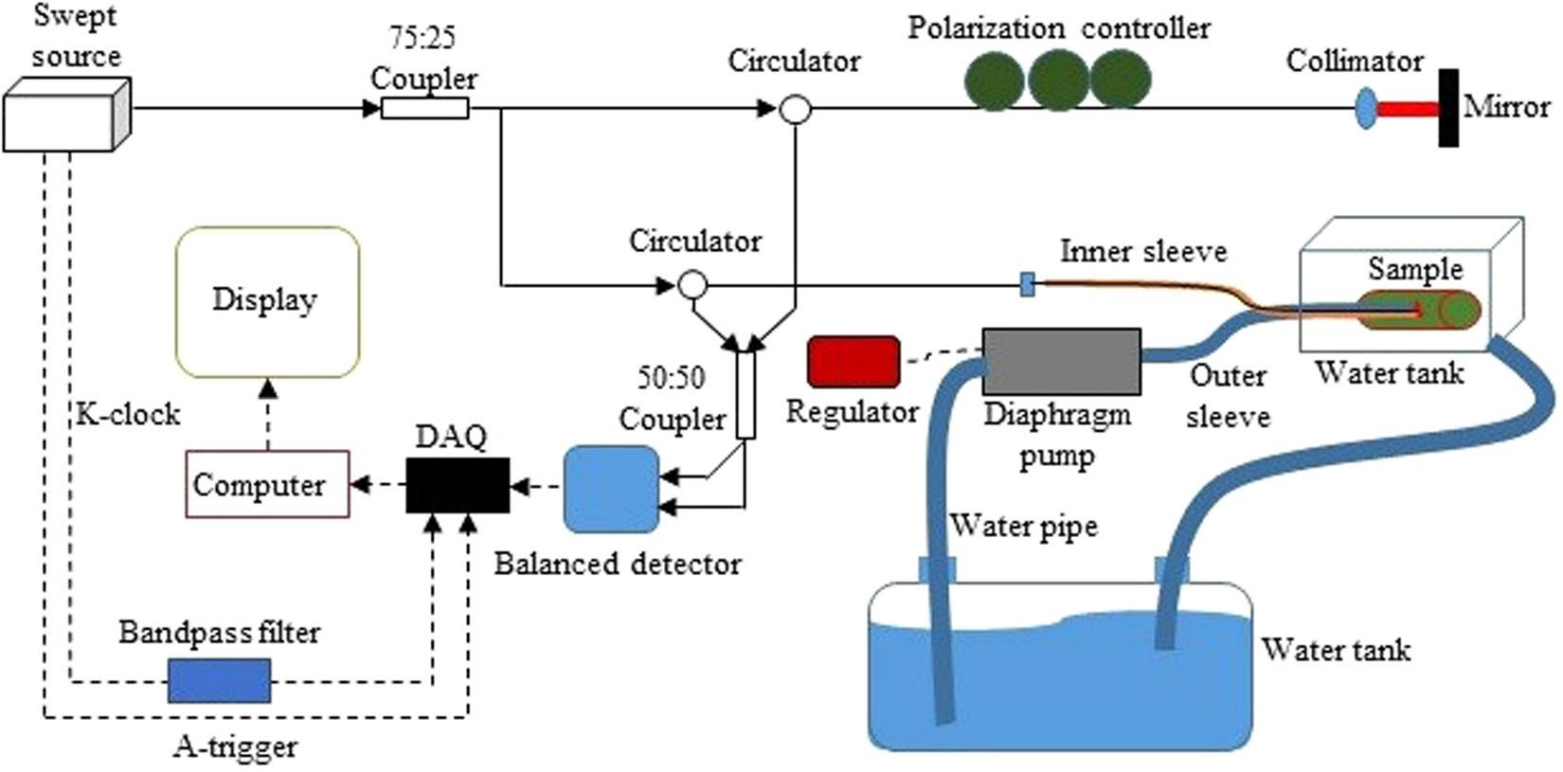
Schematic diagram of the experimental system; dotted line represents the electric signal, solid line represents the optical signal.

The schematic diagram of the IV-OCT system is shown in Fig. 5. A broadband swept laser source with a scan rate of 50 kHz, a full width half-maximal (FWHM) Δλ of 104 nm, and a center wavelength λ of 1310 nm was used as the light source. Then an axial resolution Δz of 7.3 μm in air was calculated by Δ*z* = 0.44*λ*^2^/Δ*λ*^9^. A bandpass filter was connected to the k-clock transmission cable to eliminate the DC component and the high frequency noise. The backscattered light of the sample collected from the sample arm was interfered with the light returned from the reference arm in the 50:50 coupler. The interference signal was converted into an electrical signal using a balanced detector to be processed by the computer, and then it was displayed as an OCT image. The outer sleeve of the probe was connected to a diaphragm pump, and the liquid in the large tank was injected into the outer sleeve. A regulator was used to continuously adjusts the input voltage of the diaphragm pump. The speed of the fluid was controlled by the input voltage. The test sample was placed in a small water tank. The diaphragm pump injected the liquid into the outer sleeve of the probe when the imaging was carried out. The liquid flowed from the probe to the small water tank, and it could then flow into the large water tank when the recycle use was needed.

### Rotation speed of probe

We set four gears in the low voltage range of the regulator of the diaphragm pump to test the rotation speed of the probe at different flow rate. The blood vessel phantom was an ID of 4 mm FEP tube. The liquid used in the experiment was saline. After measuring the rotation speed, the probe was placed in a graduated cylinder, and the volume of the saline discharged from the probe was measured at a certain time. The flow rate was calculated at different gears. Five tests were conducted for each gear, and the final results were the average of the flow velocity and the rotation speed at each gear, as shown in Table 1. The results of the flow rate of saline have an error less than 0.1 mL/sec. The results of the rotation speed are rounded.

**Table 1:** Results of Rotation Speed Test at Different Regulator Gear.

Gear	Flow rate of saline (mL/sec)	Rotation speed of probe (rps)
First	2.1	54
Second	3.7	103
Third	4.9	130
Forth	6.7	176

As shown in Table 1, the rotation speed of the probe is positively correlated with the flow rate of saline as expected. The speed of the probe can reach 176 rps, which is comparable to the commercial IV-OCT system with a rotation speed of about 100–180 rps^19–21^. Ref.^22^ describes the injection rate of saline can be 3–7 mL/sec with a total volume of 60 ml when CT coronary angiography is performing. Ref.^23^ describes the standard injection rates and volumes of nonionic contrast agent were as follows: 4–6 mL/sec for 8–12 mL for common carotid artery, 4–5 mL/sec for 8–10 mL for internal carotid artery, 2–3 mL/sec for 5 mL for external carotid artery, 3–4 mL/sec for 7–9 mL for vertebral artery, 6–8 mL/sec for 14 mL for subclavian artery, and 15–20 mL/sec for 30–40 mL for aortic arch. These values suggest that the proposed method can perform rapid imaging of the arteries in compliance with human safety injection standards. In addition, in order to verify the safety of the probe, we adjusted the regulator to a higher gear, and made the probe rotate under the saline flow rate exceed more than 15 mL/sec. No damage was found after two hours scanning under this condition. Combined with the results of the Table 1, we predict that the probe rotation speed can exceed 300 rps at this higher gear.

### Uniformity of the rotation speed

#### At different flow rates

**Figure 6:**
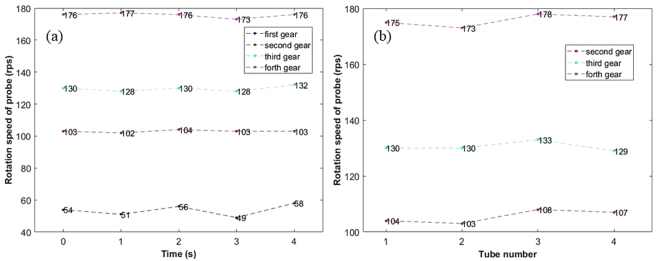
Uniformity of rotation speed of the probe. (**a**) Uniformity of the rotation speed under different gears; (**b**) Uniformity of the rotation speed in different lumens; The pipes numbered 1–4 represent FEP tubes with an ID of 4 mm, 5 mm, 7 mm, and 10 mm, respectively.

The uniformity of the rotation speed of the probe in 4 mm ID FEP tube at different flow rates is shown in Fig. 6(a). The fluctuation in the rotation speed of the probe between the adjacent times is within 2% except the flow rate at the first gear. The slightly worse uniformity of the rotation speed at the first gear was mainly due to the instability of the diaphragm pump, because we observed that the stability of the flow rate of saline was poor at this first gear. The uniformity of the rotation speed of the probe is comparable to the uniformity of the probe using a hollow ultrasonic motor in ref.^9^. It was made clear that the uniformity of the rotation speed of the probe is good.

#### In different lumens

The uniformity of the rotation speed of the probe in different lumens is shown in Fig. 6(b). In the experiment, the central axis of the probe was roughly coincident with the central axis of the different FEP tubes. As shown in Fig. 6(b), no significant difference was found of the rotational speed of the probe in different lumens. We can conclude that for the diameter of 4–10 mm lumen, the rotation speed of the probe is basically not affected by the lumen size. This indicates that the rotation speed of the probe has a good robustness in different size of lumens.

### System performance

**Figure 7:**
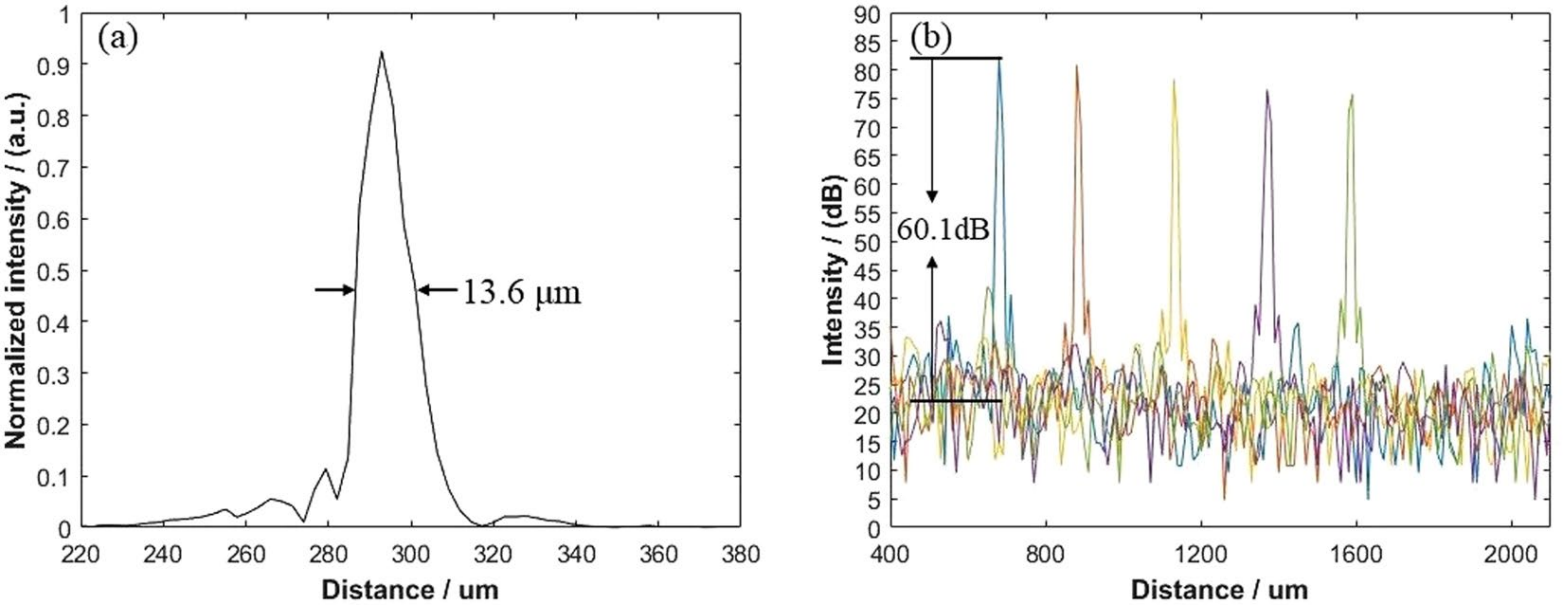
(**a**) Measured axial resolution at the depth of 300 μm. (**b**) Measured point spread function (PSF) of the system at different depths.

Experimental measurements are presented to demonstrate the detailed performance of the IV-OCT system with the probe. Figure 7(a) shows the measured axial resolution when a mirror was used as the sample. The steps of the resolution measurement are as following. First, the probe was fixed on a stage, and a mirror was used as the sample and placed on a height-adjustable stage. The mirror was placed at the working distance, which was about 300 μm out of the outer wall of the probe. Then the propeller was adjusted so that the light reflected by the right angle prism was incident perpendicularly into the mirror. The OCT image of the mirror without scanning was obtained. The full width at half maximum of the point spread function was calculated as the resolution of the system. The measured axial resolution of the system is 13.6 μm in air, and this value is larger than the theoretical value calculated from the light source of the system. The larger value in the measurement may be caused by the dispersion mismatch between the sample arm and the reference arm, and the imperfect spectrum shaping. Figure 7(b) shows the measured sensitivity of the system at different depths. Since the distance between the focal point and catheter is too short, the traditional method of inserting an neutral density filter into the beam path to attenuate the signal is not feasible^9^. To adjust the optical power, we loosed the flange connecting the sample arm and the circulator. The optical power was measured to be 8.3 mW and 68.9 μW before and after the flange was adjusted, respectively, and the attenuation was 41.6 dB. The measured signal-to-noise (S/N) ratio was 60.1 dB as shown in Fig. 7(b), which means the system sensitivity is 101.7 dB. The lateral resolution of the probe was 22 μm, which is the same with the beam waist of the fiber probe.

### Phantom and ***Ex vivo*** images

**Figure 8:**
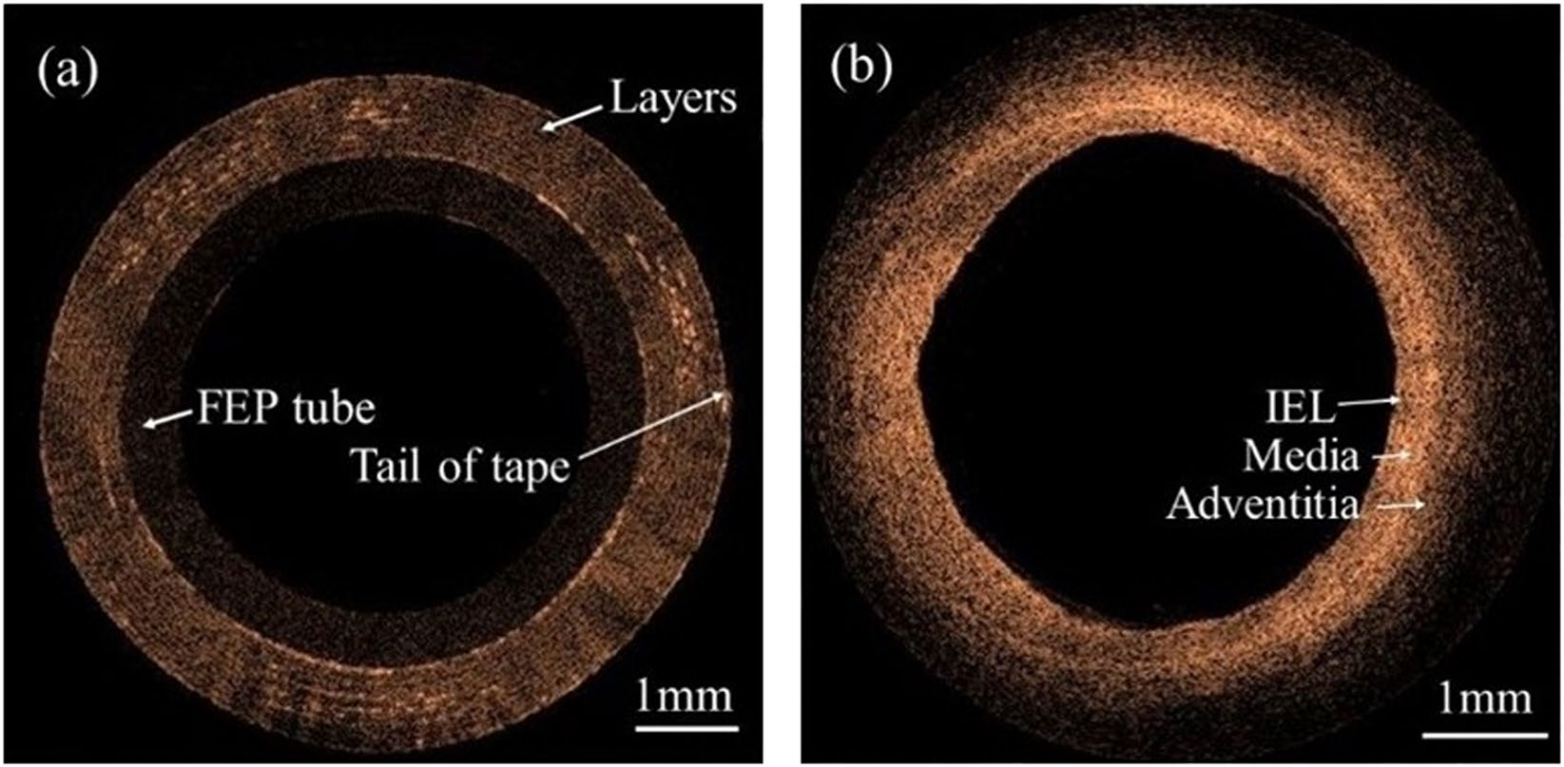
OCT images obtained with the probe. (**a**) The phantom of white tape. (**b**) *Ex vivo* porcine cardiac artery. ILE: internal elastic lamina.

Figure 8(a) shows a phantom imaged with the probe when the regulator was in the second gear. The FEP tube with a layer structure of ~60 μm-thickness white tape and its tail can be distinguished in the image. *Ex vivo* image of porcine cardiac artery is also shown in Fig. 8(b). The internal elastic lamina (IEL), media, and adventitia of the artery wall can be distinguished from the image. It is clear that all the images are unobstructed in 360 deg views with no shadow effect. The quality of image is not good due to the dispersion of the system, and this may be improved by dispersion compensation and optimize the fiber probe. A circumferential scan image consists of 485 A-scans, and the imaging rate is ~103fps which is consistent with the rotation speed of the probe in the second gear in Table 1. All images were acquired in polar coordinates in real time through the data acquisition and processing. They were post-processed in MATLAB to convert polar coordinates to Cartesian coordinates to be visualized, and preserved as the true morphology of the object.

## Discussion and Conclusion

This work is an exploratory study of IV-OCT imaging using a passive probe. Although we have demonstrated that we can obtain IV-OCT imaging with the probe based on a miniature propeller, there are a few limitations need to be overcome before this technology can be translated to *in vivo* intravascular imaging. Limited by the size of the bearing and the accuracy of the 3D printing machine, the diameter of the probe is 3.5 mm, and the length of rigid part of our probe is 14 mm. There is still room for improvement in design, and we will be committed to reducing the size of the probe in the future. Canceling the use of bearings, using a smaller GRIN lens, and nano 3D printing technology will make this improvement possible. Since nano 3D printing technology can print a micro-propeller and its supporting components with the size of hundreds of microns, a probe of the diameter less than 1 mm and rigid parts of the length less than 5 mm become possible. The rotation speed of probe still has a certain degree of non-uniformity, which may be due to the fact that the propeller and the inner casing are not completely coaxial. The use of more sophisticated assembly tools may improve the coaxiality of the probe. In addition, a pull-back device will be added to the probe system, and triggers are needed to synchronize the rotation and the data-acquisition. And then pull-back scanning will be performed to achieve real-time three-dimensional imaging.

In conclusion, we have proposed a novel passive driven IV-OCT probe based on a miniaturized propeller. The probe utilizes the fluid to flush the blood in order to rotate the propeller. This rotation in turn drives the right angle prism mounted on the propeller shaft to realize the rotational scanning. This probe achieves an unobstructed 360 deg view for circumferential imaging with a spatial resolution of 13.6 μm (axial) ×22 μm (lateral) and sensitivity of 101.7 dB. Phantom and *ex vivo* images of porcine coronary artery are provided to demonstrate the performance. The configuration of a miniaturized propeller has been proved to be a simple, robust, and feasible idea for the design of a distal scanning probe. The probe is fabricated with biocompatible materials, and the low cost of the probe and good experimental results make it clear that the probe has a great potential for clinical application.

## Methods

### Assemble the probe

A hole was opened about 70 cm away from the end of the outer sleeve, and the inner sleeve was inserted into the hole. The end of the insert part of inner sleeve extended beyond the end of the outer sleeve by about 1.5 mm. The probe looked like a “Y” shape after the insertion. In order to reduce production costs and speed up the production cycle, the miniaturized propeller was made from 3D printing using photosensitive resin. The end of the propeller shaft was polished to a diameter of about 0.6 mm and then it was inserted into the micro bearing to tightly engage with it. The right angle prism of 0.5 mm size was then glued on the shaft of the propeller. The customized fiber probe was inserted and glued with the inner sleeve, and the end of the fiber probe was about 1.5 mm away from the end of the inner sleeve. Then, the right angle prism, the micro bearing, and the propeller that have been assembled as an integral part were inserted into the end of the inner sleeve, and the outer ring of the micro bearing was glued with the inner wall of the inner sleeve.

Two stainless steel wires of 14 mm length and 0.2 mm diameter were used to make the protection cover. Each steel wire was bent into a “U” shape to have two steel wire columns, so that the protection cover had four steel wire columns in total, and the angle between the two adjacent steel wire columns was 90 deg. The end of the protection cover was glued with the outer wall of the inner sleeve to completely wrap the propeller, and the junction of two stainless steel wires was also glued to fix them.

Then, three stainless steel wires with 8 mm length and 0.3 mm diameter were adhered equidistantly to the outer wall at the end of the inner sleeve, and the end of the steel wires was about 3 mm away from the end of the inner sleeve.

Finally, the inner sleeve and the outer sleeve were fixed together with glue at the opening hole of the outer sleeve.

### Rotation speed test

To measure the rotation speed of the propeller, the probe was inserted into the FEP tube with an ID of 4 mm which was fixed to a small base attached to the bottom of the tank, and the axis of the probe was approximately coincident with the axis of the FEP tube. The probe was also fixed on a similar small base after being inserted into the FEP tube. The tank was filled with saline which completely submerged the probe and the FEP tube. A photoelectric receiver was placed near the water tank. When the laser emitted from the probe was incident on the photoelectric receiver after passing through the FEP tube, the water, and the water tank wall, a high level signal of about 5 V was outputted, and an oscilloscope was connected to the output of the photoelectric receiver to receive the signal and record it. The oscilloscope receives and records a high level signal at each turn of the propeller. An algorithm that automatically calculates the propeller speed by detecting the frequency of the high level signal was developed by using MATLAB. Since the photoelectric receiver detected a laser in wavelength range of 400–1000 nm, a laser light of a single wavelength of 532 nm was used to test the rotation speed of the propeller. The 4 mm ID FEP tube was replaced with other FEP tubes, and a consistent procedure of the speed test was executed to test the rotation speed of the probe in different lumens.

### Phantom imaging

The phantom was made by coiling several layers of white tape on a FEP tube with a length of 15 mm, an ID of 4 mm, and a wall thickness of 0.5 mm. The phantom was then fixed to a small base attached to the bottom of the tank. The probe was also fixed on a small base which was stuck to the bottom of the tank after the probe was inserted into the phantom. The phantom was imaged after the tank was filled with saline, which completely submerged the probe and the phantom.

### *Ex vivo* porcine cardiac artery imaging

The fresh porcine heart used in the experiment was purchased from the slaughterhouse, and it was frozen in a 4 C degree freezer during the transportation of about 3 hours. After the imaging system was set up, a 3 cm section of the cardiac artery with an outer diameter of about 4 mm was isolated from the thawed heart. The probe was passed through the excised cardiac artery, and then it was placed in the water tank filled with saline for imaging.

### Data availability

The datasets generated during and/or analyzed during the current study are available from the corresponding author on reasonable request.
